# Philadelphia Hospital

**Published:** 1838-12-05

**Authors:** 


### CLINICAL LECTURES.

### PHILADELPHIA HOSPITAL.

#### ON PLEURISY, PERICARDITIS, ETC.

*Saturday, Nov.* 24.—Dr. Gerhard said: You may remember that a patient, labouring under ascites, was presented to you about a fortnight since. The nature of the disease was then stated, and it was then mentioned that the prognosis was extremely unfavourable. The patient had been previously attacked with external fungus hæmatodes. The tumour appeared on the cranium, and was readily removed by Dr. Horner. Diarrhœa supervened during his residence in the surgical ward; he was finally transferred to the medical ward, was cured and discharged; went to work, and continued well for two months, when he was attacked with chills, sweats, and, finally, pains in the back and abdomen, which gave him much uneasiness for a month previously to his entrance. The swelling of the abdomen was perceived nearly a week previously.

He has now been in the hospital almost three weeks, and has been subjected to various remedies without advantage. Frictions of camphorated mercurial ointment were made over the abdomen until ptyalism took place. Diuretic drinks, such as an infusion of juniper berries, a solution of supertartrate of iron, and of cream of tartar, have also failed. We have, finally, placed the patient upon squills and digitalis, in the proportion of two grains of the former and two-thirds of a grain of the latter three times daily. We have attempted the sudorific and purgative plans of treatment, but they were not persevered in sufficiently long to ascertain whether any benefit would be obtained from them.

On the failure of these means, the operation of paracentesis seems absolutely necessary, less with the hope of restoring the patient to health, than of affording him a degree of comfort which cannot be obtained while the abdomen remains so much distended. If the operation is not performed, his death is certain within a short period; if it be performed, he will certainly be relieved of the distension and of most of the pain which he now suffers; but, on the other hand, he runs considerable risk from the occurrence of acute peritonitis, an accident which is by no means rare after tapping, particularly in the advanced stage of the disease which this patient has reached. Under all these circumstances, the most advisable course was to propose, but not to urge, the operation upon the patient; a fortnight ago he objected, but last evening he requested that it should be performed.

The prognosis which is so unfavourable in the case, depends,—1st. Upon the fungus hæmatodes, which, as you well know, is a constitutional disease, and, although extirpated upon the exterior, has, probably, attacked an internal organ—the liver. 2d. Upon the disease having followed a chronic diarrhœa, which was, in some measure, dependent upon his original constitutional disorder. When ascites comes on, with flying pains in the abdomen, after the cessation of diarrhœa, we usually regard it as arising from a chronic peritonitis complicated with tubercles of the serous coat. But as he was already affected with a chronic disease of a different nature, the probability of the previous occurrence of tuberculous inflammation is by no means equally great.

[Upon consultation with Dr. Gibson, the operation was performed by him in presence of the class in the usual manner, but only about two-thirds of the whole quantity of liquid contained in the abdomen was removed. It was thought more prudent not to take away the whole quantity thus suddenly from a patient in this exhausted condition.]

I shall now state in what condition the patients remain, who were presented to you on last Saturday. One, you may recollect, was a young man, who had entered the hospital immediately before the lecture. He had a dry, red tongue, much nausea and vomiting, with great tenderness at the epigastrium, there was a little cough, some dyspnœa, and considerable fever. The case was mentioned as one of gastritis; and the patient was directed to use demulcent drinks, to diet, and to be cupped over the epigastrium. On Monday the symptoms of gastritis had diminished, but there was a suspicious eruption of little reddish points over the greater part of the body. On Tuesday these elevations were vesicular, and the fever had completely ceased. On Wednesday the vesicles had become purulent, and the patient was transferred to the City Hospital, as a well-marked case of varioloid. This case is highly interesting to you; you witnessed the great distress of the patient, the very evident gastritis, and the high fever which accompanied the local symptoms, the limited influence of treatment, and the rapid subsidence of the symptoms as soon as the eruption appeared. This is almost a peculiarity of varioloid; the premonitory fever is often quite as great, sometimes even greater, than in variola; but the moment the eruption comes out, an entire calm follows the great disturbance of all the organs, and the patient is well as far as the general symptoms are concerned. I might pursue the analogies of this case much further, and speak of various forms of eruptive fever, characterized by intense general symptoms, and partial or complete subsidence of them as soon as the characteristic eruption appears. In other forms of eruptive fever, there is, however, usually something characteristic from the very beginning; but in variola, and indeed in all variolous diseases, there is no absolute connexion between the symptoms which precede and remain after the eruption. You may entertain a suspicion of the true state of the case, but you can never be sure of it; the suspicion becomes stronger, if an epidemic happens to prevail; but when we see only sporadic cases, we are frequently at fault. In these cases you must still exercise your powers of diagnosis; and if you are not certain of the future progress of the disease, you may, at any rate, with great advantage, treat the gastritis, or the cerebral irritation, and thus relieve your patient, and perhaps render the course of the disease decidedly milder.

The symptoms which may give rise to a probable diagnosis before the appearance of the eruption, are the violence of the fever, the local signs of gastritis, and the intense pain in the head and neck, which are more severe than in most febrile diseases.

I shall now proceed to demonstrate to you another case of disease, which has just entered the hospital:

Thomas Riley, aged twenty-three years, a single man, entered the hospital November 22d, 1838. Born in Ireland; in this country for four years; a labourer; at work as an ostler for the last sixteen months, during which time he was quite well; sober. He was taken ill six weeks ago; does not recollect the day. Was taken with pain at the lower extremity of the sternum; had been driving an omnibus for two days during a hard and steady rain, at the end of which time he was taken ill. Pain continued at the point first attacked. Oppression and cough followed; first the dyspnœa, and the cough about two weeks before his entrance; the pain has ceased for last four days, but the oppression continues—a little lighter; cough is better; not able to lie on his back always; at times obliged to sit up until morning. As soon as dyspnœa occurred, a blister was applied to the right side of his chest, three and a half weeks before his entrance, when he was able to lie on that side; could never lie for any time on left side; prevented by increase of oppression; cough most at night; expectoration whitish, thin; sleep sound, no dreams; had pain at the right side also for five days, beginning about two and a half weeks after that at the sternum, which had ceased five days after its occurrence, on the application of a blister; no swelling of the face, but has had swelling of the feet for five weeks, nearly stationary; palpitations not distinct; scarcely recollects them; gave him little trouble; had them on walking up and down stairs; kept his bed after the first three days, and had no palpitations, but had them on rising and walking up stairs after three weeks; chill on the third day, and again repeated the same night; felt rather better afterwards; sweats chiefly at night; little fever; thirst great; appetite good; bowels costive; was quite well before the present attack; no shortness of breath, palpitations, nor epistaxis; (never had the latter but twice;) never had rheumatism or syphilis. Was attended by a physician; bled once, and took some purging medicine; no one saw him for the last three weeks, during which time he applied a blister to his side, and drank milk.

*Present state;* Nov. 23d.—Well made; dark complexion; slight emaciation. Feet and legs a little œdematous; decubitus dorsal, sensibly elevated; nostrils dilating; lips a little purple; no flush of the cheeks; intellect very clear; no cephalalgia; strength very feeble; respiration thirty-two; high, quick, and noisy inspiration; costal; no pain on breathing; it has ceased for four days past; pulse one hundred and twenty-eight; bis feriens; easily compressed, a little irregular; almost no expectoration; cough short, dry; voice clear; skin warm; sweating cool, slight; no chill; tongue whitish, moist; appetite good, less than in health; costive, no discharge for three days; abdomen tympanitic at the lower portion.

*Thorax.*—Manifest distension of the right side; percussion on the left side, clear throughout; posteriorly, on the right, complete flatness; a little less perfect at summit of lung, most perfect laterally; anteriorly, right side, flatness nearly perfect to clavicle; left side clear, except at the præcordial region, where the flatness extends an inch to the left of it from fourth rib down; flatness from right side extends to the left margin of the sternum; respiration posteriorly, at the summit of the right side, tubal; at the middle third, of the same character, a little less intense; in lower third, still less strong; no distinct vesicular sound; in the axilla, the same feebleness of respiration, and bronchial; anteriorly, it is the same at the right side, at the summit tubal; in descending the same character continues, but gradually diminishing and replaced by very feeble respiration. Voice—at the summit of right side, strong bronchophony; at middle third, and especially towards the axilla, strong egophony, which gradually becomes less intense in descending; anteriorly, at summit, bronchophony; egophony, observed in lower two-thirds. Left lung, at the summit, posteriorly, respiration vesicular, but the bronchial sound is conducted from opposite lung; below, the respiration is vesicular and puerile; in the axilla, the same character observed, and at upper part of left side anteriorly. Sounds of heart both heard; impulse exaggerated.

℞. Tinct. digitalis ʒj.

Spiritus ether. nitros. ℥i.

Pulv. gum. acac. ʒj.

Aquæ menth. p. ℥iij.

M. ft. mist.

To begin to-morrow morning, half an ounce, four times a day.

℞. Vini sem. colchic. ʒj.

Magnes. sulphat. ℥ss.

Magnes. ustæ ʒj.

Aq. menth. p. ℥iv.

M.

Take one half, and if not purged, take second half at end of three hours. Gruel, bread, tea, &c.

In this case the problem afforded for diagnosis, is a very simple one. We may ascertain the nature of the disease by the general symptoms and local physical signs. First, of the general signs; these are usually sufficient for the diagnosis of thoracic diseases one from another, but not for distinguishing the variety, or for ascertaining the progress of a particular disease. The method of using the general or functional signs in diagnosis, depends, in part, upon our knowledge of the functions of a particular organ, which are necessarily more or less altered as soon as the organ becomes seriously affected; and in part from the absence of the signs indicative of suffering in those organs of the body which remain healthy. The latter mode is termed diagnosis by way of exclusion, and is extremely useful when a diseased organ does not present very marked symptoms. We then run over in our minds the symptoms of other diseases more or less similar in some respects to those which the patient before us presents, and after we have found that they are wanting we may arrive with great certainty at the knowledge of the real disease. This method of diagnosis requires, of course, a certain knowledge of pathology,—that is, we must know positively what are the signs of various diseases, before we can undertake to say that they are absent in a particular case.

You will understand this better by the table, which I will now draw upon the black board; I arrange the symptoms belonging to particular organs in separate columns upon the board, and run lines across those columns which correspond to organs not presenting any important functional disorder.

| HEAD. | NECK. | THORAX. | ABDOMEN. |
|---|---|---|---|
| \ | \ | Cough slight.<br>Expectoration catarrhal, white<br>Dyspnœa, great.<br>Sharp pain in right axilla on breathing or coughing. | \ |

You see that I have stricken out the symptoms belonging to all the cavities, except the thorax. I find that both the cough and expectoration are insignificant, but we have, on the other hand, as positive symptoms, the dyspnœa and lancinating pain in the lower part of the thorax. We have, then, a right to conclude that there is no important lesion of any organ, except those belonging to the thorax, and that in the thorax neither the mucous coat of the bronchial tubes, nor the parenchyma of the lungs are affected to a sufficient degree to produce either characteristic cough or expectoration. There remain two symptoms, the acute pain, and the dyspnœa; these both belong to pleurisy, which affects the right side only.

I have purposely omitted the symptoms belonging to the circulating organs, although they properly belong to the thorax. It would have complicated the diagram to have introduced them at first. Besides, these symptoms are not exclusively confined to the movement of the heart or the passage of the blood through the large vessels, but extend also to the capillary system, and thus include the whole body. I will, therefore, write them separately from the others, in a single line:

Edema of lower extremities. Pulse irregular, at least one hundred and thirty.

These signs are, in part, characteristic of a disease of the heart, and in part belong to every case in which there is much febrile excitement. The frequency of the pulse is of little importance in itself; it becomes so when combined with its irregularity, and then furnishes probable evidence of a disease of the heart. Edema of the limbs in an acute disease, is also another very probable evidence of a disorder of the central organ of circulation, and scarcely occurs at so early a period from any other cause. We may, therefore, regard the evidence as conclusive, that there is a real organic disease of the heart itself. The nature of the acute disease is known by other evidence. We ascertain from the patient that he was quite well previously to the present attack, had neither palpitation, oppression, nor other evidence of chronic heart disease. Hence we are enabled to state that the disease is acute, and connected, in all probability, with inflammation.

By reference to another law of pathology, the diagnosis is rendered still more precise. We have ascertained by an examination of the symptoms that the patient labours under pleurisy of the right side; now, we know that inflammation of the pleura and pericardium frequently coincide, and that, when there is pleurisy, if the heart participate in the disease, it is by inflammation of its serous coat. We might, therefore, conclude, that the evidence in this case is thus far sufficient to direct us to the nature of the disease, and its complications. Still this evidence is only probable; it is more probable for them who have great familiarity with disease, than for those who are not sufficiently acquainted with its phenomena to exclude from the calculation necessary for the diagnosis of a particular affection, symptoms which are not present, than it is for physicians who are not familiar with the whole circle of pathology. If we wish to render the diagnosis still more certain, we are obliged to resort to the local signs, as I shall presently demonstrate to you.

In the first place, we examine the chest of this patient. By the eye you may ascertain—even those of you who are at the distance of several feet from the patient—that the right side of the chest is excessively dilated, and its natural conformation entirely changed. Measurement would also prove the same fact, but we can ascertain the existence of moderate degrees of dilatation much more readily by the eye, than by measurement, which is only useful in the most obvious cases. The dilatation arises from the liquid effused into the right pleura. The same liquid compresses the lung, forces out the air, and prevents the clear resonance on percussion. The third kind of physical signs are those furnished by the respiration and voice: in the upper half of the right lung the respiration is decidedly bronchial, and, in the lower half, it is nearly absent; that is, the compression of the pulmonary tissue is sufficient at the lower two-thirds to prevent the entrance of air into the pulmonary tissue, while at the upper portion of the lungs the respiration still continues through the large tubes, which are not completely obliterated by the pressure. It is bronchial instead of vesicular, because the vesicular structure is compressed much more readily than the larger tube; hence the vesicular murmur is destroyed more quickly than the bronchial respiration.

The voice yields signs equally characteristic; at the summit of the lungs we have decided bronchophony, and at the middle portion, in a zone extending along the inferior angle of the scapula to the anterior part of the thorax, the resonance of the voice is vibrating, or egophonic—a sign pathognomonic of effusion into the pleura, the liquid through which the voice is transmitted causing the peculiar vibration.

Now that I have demonstrated to you the physical signs of pleurisy, I have but a word or two to say respecting those of pericarditis. The dulness on percussion at the præcordial region, the feeble impulse of the heart, and the distance at which its sounds are heard, are the signs observable in the patient, and, with the general symptoms, are quite characteristic of this disease. Still, in most cases of pericarditis, the physical signs are more evident; as you may readily understand, when you remember that the disease had commenced in the pericardium some weeks since.

Of the treatment and prognosis of this case, I shall afterwards speak.

Dr. Gibson introduced the following cases:

*First.* A case of entropium, or trichiasis. After making some general observations upon this disease, the Doctor proceeded to operate, by removing an oval portion of the superfluous skin of the eyelid, with the forceps and scissors. The edges of the wound were drawn together by two stitches of the interrupted suture, and the whole covered with a light dressing.

*Second.* A case in which the prepuce was so enlarged, as to form a troublesome tumour on the inferior and lateral parts of the glans penis. This case also presented a specimen of the *ulceration en arcs* of Rayer, or the horse-shoe ulcer of the Dublin schools. Dr. Gibson dissected away the hypertrophied mass; slight hæmorrhage occurred, which was arrested by the application of ligatures, simple dressing, and a bandage to the penis.

*Third.* A case of hydrocele, of fourteen years' standing. Dr. Gibson operated, by puncturing the sac, evacuating the fluid to the amount of ℥xij., and introducing a seton.

*Fourth.* A case of chronic mortification of the right foot and ankle, occurring in a female aged twenty-nine. A black spot upon the instep was first noticed November 19th, five days since. This spot has gradually increased until the present time, involving the whole foot and ankle. Patient very weak, and sinking rapidly. Dr. Gibson ordered stimulants, a full diet, and fermenting poultices to part.

*Fifth.* A case of ascites, from Dr. Gerhard's wards, by whom the history of the case was given. Dr. Gibson performed the operation of paracentesis abdominis, and drew off about two gallons of fluid.

[We have not given at length Professor Gibson's clinical remarks on the cases to-day introduced, but refer our readers to full reports on these topics, published during the last clinical season. Lectures on subjects not previously noticed, the progress of the course will soon enable us to present in detail.—Eds.]

